# Gene set enrichment analysis for multiple continuous phenotypes

**DOI:** 10.1186/1471-2105-15-260

**Published:** 2014-08-03

**Authors:** Xiaoming Wang, Saumyadipta Pyne, Irina Dinu

**Affiliations:** School of Public Health, University of Alberta, Edmonton, AB T6G 1C9 Canada; CR Rao Advanced Institute of Mathematics, Statistics and Computer Science, Hyderabad, AP 500046 India; Public Health Foundation of India, Delhi, India

**Keywords:** DNA microarrays, Gene expression, Linear combination test, Nonlinear combination test, Gene-set analysis

## Abstract

**Background:**

Gene set analysis (GSA) methods test the association of sets of genes with phenotypes in gene expression microarray studies. While GSA methods on a single binary or categorical phenotype abounds, little attention has been paid to the case of a continuous phenotype, and there is no method to accommodate correlated multiple continuous phenotypes.

**Result:**

We propose here an extension of the linear combination test (LCT) to its new version for multiple continuous phenotypes, incorporating correlations among gene expressions of functionally related gene sets, as well as correlations among multiple phenotypes. Further, we extend our new method to its nonlinear version, referred as nonlinear combination test (NLCT), to test potential nonlinear association of gene sets with multiple phenotypes. Simulation study and a real microarray example demonstrate the practical aspects of the proposed methods.

**Conclusion:**

The proposed approaches are effective in controlling type I errors and powerful in testing associations between gene-sets and multiple continuous phenotypes. They are both computationally effective. Naively (univariately) analyzing a group of multiple correlated phenotypes could be dangerous. R-codes to perform LCT and NLCT for multiple continuous phenotypes are available at http://www.ualberta.ca/~yyasui/homepage.html.

**Electronic supplementary material:**

The online version of this article (doi:10.1186/1471-2105-15-260) contains supplementary material, which is available to authorized users.

## Background

Microarray data analysis at an individual gene level usually leads to a large list of significant genes, even after multiple comparison adjustment has been made. The process of trying to interpret such a large list of genes is difficult. Molecular biologists have put together lists of genes grouped by function, such as biological pathways or sets of genes. Various pathways or gene sets databases have been compiled, for example, Kyoto Encyclopedia of Genes and Genomes (KEGG) [1, 2], Gene Ontology [3], Biocarta [4] and Molecular Signature Data Base [5]. There has been a shift in focus from gene level analysis to pathway level, or gene set level, with many Gene Set Analysis (GSA) methods being proposed in the past decade. The most popular one is Gene Set Enrichment Analysis (GSEA) [6]. Extensive reviews and methodological discussions were given by Goeman and Buhlmann [7] and Nam and Kim [8].

While GSA methods on a single binary or categorical phenotype abounds, little attention has been paid to the case of a continuous phenotype, and there are no methods to accommodate correlated multiple continuous phenotypes. Such correlated continuous variables are measured routinely in many important clinicopathological observations such as lung functions, tumor size or measurements of marker proteins. A naïve approach to analyzing such data with existing GSA methods would be to categorize the continuous phenotypes into two or more discrete classes, as well as analyze the multiple correlated phenotypes univariately, i.e., one at a time. Such artificial categorization and univariate analyses may lead to less efficiency in gene-set analysis and even cause inaccurate identification of significant gene sets, especially if the multiple phenotypes exhibit relatively higher correlations.

There is an important methodological distinction between the competitive and self-contained GSA approaches [6, 7]. For a binary phenotype, e.g., competitive methods use gene permutation to test whether or not the association of the phenotype with a gene set is similar to its association with the other gene sets (the “Q1 hypothesis”), while self-contained methods employ sample permutation to test the equality of the means of the two vectors of gene-set expressions which correspond to the two phenotype groups (the “Q2 hypothesis”). Here, we focused on the self-contained methods. Unlike the gene permutation strategy, sample permutation preserves correlation structure within gene sets and correlation structure within phenotypes -- a key property that we wish to fully take advantage in the proposed GSA methods.

To the best of our knowledge, although correlations among genes in gene sets have long been observed, correlation structure was considered only in a few GSA methods. These were the modified Hotelling’s T^2^ test for categorical phenotype [9], and the linear combination test (LCT) for binary phenotype [10] and for continuous phenotype [11]. It has been realized that incorporation of correlations among gene expressions in a GSA approach can significantly improve efficiency of the analysis [9]; however, it could also spell a heavy computational burden. The linear combination test was designed to incorporate correlations among gene expressions while overcome the computational burden. In the case of binary phenotype, it has been showed that LCT was much more computationally efficient than the modified Hotelling’s T^2^ test and approximated its superior power very well [10]; in the case of continuous phenotype, it has been showed that LCT was superior in power to the other GSA methods under compare [11].

We propose here an extension of LCT to its new version for multiple continuous phenotypes, incorporating correlations among gene expressions of functionally related gene sets, as well as correlations among multiple phenotypes. Further, we extend the new method to its nonlinear version, referred as nonlinear combination test (NLCT), to test potential nonlinear association between gene sets and multiple phenotypes, especially recommended for analyzing relatively larger microarrays. The extension strategy can also be used for other GSA tools for continuous phenotype/phenotypes, such as Global Test [12]. The rest of the article is organized as follows. In section 2 we give detailed derivations of the two proposed GSA methods. In section 3, we used a simulation study to show the practice aspects of these two proposed methods using various settings on sample size, gene-set size, and correlation level among genes and among phenotypes. Section 4 presents the performances of the proposed methods on a real gene expression microarray data from prostate tumor samples of African-American prostate cancer patients [13].

## Method

### Linear combination test for multiple continuous phenotypes

Consider a microarray study on *n* subjects, with measurements on expressions of a predefined set of *P* genes *X* = (*x*_1_, …, *x*_*p*_)^*T*^ and measurements on a group of *q* continuous phenotypes *Y* = (*y*_1_, …, *y*_*q*_)^*T*^. Suppose columns in both *X* and *Y* are centered and scaled across the subjects. We are interested in testing whether there is a significant linear relationship between the gene set *X* and the group of phenotypes *Y*. The null hypothesis to be tested is that expressions of the genes in the predefined gene set *X* are linearly independent with the phenotypes *Y*. The multivariate null hypothesis can be expressed linearly and univariately as

*H*_*0*_*: There is no association between any of the linear combinations of x*_1_, …, *x*_*p*_*and any of the linear combinations of y*_1_, …, *y*_*q*_*.*

To test the linear relationship, let *Z*(*X*, *A*) = *a*_1_*x*_1_ + ⋯ + *a*_*p*_*x*_*p*_ be a linear combination of *x*_1_, …, *x*_*p*_, and *Z*(*Y*, *B*) = *b*_1_*y*_1_ + ⋯ + *b*_*q*_*y*_*q*_ a linear combination of *y*_1_, …, *y*_*q*_, where *A* ∈ *R*^*p*^ and *B* ∈ *R*^*q*^ represent the coefficient vectors of *a*_*i*_'s and *b*_*j*_'s, respectively. For given coefficient vectors *A* and *B* of the combination coefficients, we can focus on testing whether the combination *Z*(*X*,*A*) is associated with the combination *Z*(*Y*,*B*). This is a classical correlation test and a commonly used test statistic is based on measuring the Pearson correlation between *Z*(*X*,*A*) and *Z*(*Y*,*B*), i.e.  = (*Z*(*X*,*A*),*Z*(*Y*,*B*)). If both *X* and *Y* are normally distributed, then the statistic  follows a Student's t-distribution with degrees of freedom *n* -2 under the null hypothesis [14]. This also holds approximately if the observed values are non-normal, provided sample size *n* is large enough [15].

For testing the null hypothesis H_0_, we consider the linear combinations of *x*_1_, …, *x*_*p*_ and *y*_1_, …, *y*_*q*_, exhibiting the highest correlation, i.e. choosing coefficient vectors *A* and *B* to maximize the Pearson correlation between *Z*(*X*,*A*) and *Z*(*Y*,*B*). This leads to the proposed new version of the linear combination test (LCT) for multiple continuous phenotypes
1

The old version of LCT for single continuous phenotype [11] is a special case of it.

Let *Σ*_*XX*_ = cov(*X*, *X*) be the covariance matrix of *X* whose (*i*,*j*) entry is *σ*_*ij*_ = cov(*x*_*i*_, *x*_*j*_); and similarly, let *Σ*_*YY*_ = cov(*Y*, *Y*) and *Σ*_*XY*_ = cov(*X*, *Y*) be the covariance matrix of *Y* and the covariance matrix between *X* and *Y*. The above statistic can be written as
2

When the dimension of *X* and/or dimension of *Y* are high, singularity of *Σ*_*XX*_ and *Σ*_*YY*_ have to be taken care of very carefully, especially when the size of the gene set is larger than the sample size, i.e., *p* > *n*. A possible remedy for the singularity problem is to employ the shrinkage technique proposed by Schafer and Strimmer [16], and replace *Σ*_*XX*_ and *Σ*_*YY*_ with their shrinkage versions, namely,  and . More specifically, the (*i*,*j*) entry of the shrinkage covariance matrix  is given by , with shrinkage coefficients *γ*_*ij*_ = 1, if *i* = *j*, and *γ*_*ij*_ = *ρ*_*ij*_ min(1, max(0, 1 − λ*)), if *i* ≠ *j*, where *ρ*_*ij*_ is the sample correlation between *x*_*i*_ and *x*_*j*_, and the optimal shrinkage intensity can be estimated by . Based on this shrinkage strategy, we get the shrinkage version of the test statistic
3

The computational cost on calculating (3) has to be taken into consideration, since the right hand side is a nonlinear programming problem involving *p* + *q* parameters. The computational price can be very high for maximizing directly the right hand side of (3), especially when permutation is used for calculating p-value of the test. To address the computational efficiency problem, we adopt a strategy of using two groups of normalized orthogonal bases, instead of using the original observation vectors of *X* and *Y*. We perform eigenvalue decompositions for the two shrinkage covariance matrices,  and , and obtain two groups of orthogonal basis vectors,  and . The test statistic in (3) can further be rewritten as
4

where ,  and  is the covariance matrix between  and , with its (*i*,*j*) entry being .

The optimization problem in (4) can be solved in two steps. Firstly, for a given *β*, find the optimal *α*, which is proportional to ; secondly, substitute the optimal *α* into (4), and find the global optimal *β*, which is proportional to the first eigenvector of the matrix  corresponding to the largest eigenvalue. We note that the value of *T*^2 *^ equals to the largest eigenvalue of either the *q* × *q* matrix or the *p* × *p* matrix . The cost for getting the largest eigenvalue is low, providing min(*p*,*q*) is not big.

The computation advantage is obvious when sample permutations are used to calculate p-value of the test. Since sample permutation changes neither the correlation structure within gene sets nor the correlation structure within phenotypes, so that we don’t need to repeat the same eigenvalue decompositions of the two shrinkage covariance matrices in (3) for the permuted data, but only for the original one. In fact, after performing the eigenvalue decompositions for the two shrinkage covariance matrices  and  and creating two groups of orthogonal basis vectors  and , permutations can be done only on  directly, instead of on the original phenol-type *Y*.

### Nonlinear combination test for multiple continuous phenotypes

The proposed LCT method assumes a linear relationship between the genes in a gene set and the phenotypes. So do almost all the *self-contained* GSA approaches that have been proposed in the literature. The reason for us to focus on testing linear relationship is mainly for simplicity of the method. When we have access to limited data points, a simpler approach could be more reliable than a complex/flexible one. If a larger sample size is available or if there is evidence that the relationships between gene sets and phenotypes could be non-linear/non-monotone, we may consider relaxing the linearity assumption, and testing more general null hypnoses, i.e.,

*H*_0_^*^: there is no relationship between genes in the gene set and the phenotypes.

The linear combination test proposed can be easily adapted to test nonlinear relationships between genes in a gene set and phenotypes, by using nonparametric techniques. The main idea here is to apply a non-linear transformation to the vectors of genes *X*, then use linear test methods to check if there is a significant linear relationship between the non-linear transformation of *X* and the phenotypes *Y*. This strategy is similar to that of ‘basis expansion’ which is widely adopted in regression/discrimination analyses [17]. Some widely used non-linear transformations are polynomial transformations of single or multiple genes to achieve higher-order Taylor expansions; cubic splines or wavelets transformations of single genes. We note that the same transformation strategy can be applied to the phenotypes *Y*. We prefer to leave Y untransformed to avoid higher flexibility of the method, which requires larger sample size as well as higher computational costs. In our NLCT test method used in the simulation study and the real microarray example study, we transform each gene in a gene set to a natural cubic spline with the degree of freedom set at 5.

### Simulation study design

Our simulation study was designed to check performance of both LCT and NLCT methods. More specifically, we focused on the type-I-error performance and the power performance of the proposed tests, by varying gene-set size, sample size, and correlation levels among genes and among phenotypes.

We describe below our simulation study design. For each gene-set of size *p*, a gene expression matrix ***X***_*n* × *p*_ was generated from a multivariate normal distribution. The correlation between each pair of genes was set at *p*, with values of 0.0, 0.3, 0.6, or 0.9. For each gene set, a group of continuous phenotypes of size *q* were generated from the following multivariate linear model,
5

where ***β***_*p* × *q*_ is a coefficient matrix, and ***ϵ***_*n* × *q*_ the error matrix generated from a multivariate normal distribution. The correlation between each pair of the errors was set at *p* so that each pair of the columns in *Y* is correlated with correlation *ρ*. In the null model, used to check the size of the tests, we set all entries of ***β*** to 0, so that columns in ***X*** are not correlated with columns in ***Y***. In the alternative model, used to check the power of the tests, we randomly selected five rows and three columns of the coefficient matrix, and set the corresponding fifteen entries to a common value *μ*, ranging from 0 to 5, with an increment of 0.25. The rest of the entries in the coefficient matrix were set at 0. We noted that the five selected columns of ***X*** are correlated with the three selected columns of ***Y***, and that the correlation increases with *μ*. We used various sample sizes and gene-set sizes, including large *p* and small *n*, a scenario which is common in gene-set analysis. Because the LCT and NLCT procedures are in fact tests of correlation between two groups of variables, ignoring which is gene group and which is phenotype group, we set q as fix and changed p in the simulation design. The simulation data were replicated 1,000 times in each model. The p-values were calculated based on 1,000 permutations.

## Results

### Simulation study

The type I errors are similar across the LCT and NLCT methods (Table 1), with those of LCT more closer to nominal level of 0.05, indicating lower sample size could lead to relatively higher type I errors of NLCT compare to LCT.

**Table 1:** **Type I errors of the multiple version of LCT and NLCT GSA methods, with dimension of the multiple phenotypes set at** ***q*** **= 10**

Method	ρ	*n* = 20			*n* = 50		
		*p* = 20	*p* = 50	*p* = 100	*p* = 20	*p* = 50	*p* = 100
**LCT**	0.0	0.050	0.047	0.047	0.052	0.043	0.047
	0.3	0.051	0.053	0.050	0.055	0.045	0.043
	0.6	0.050	0.054	0.042	0.051	0.042	0.048
	0.9	0.053	0.052	0.044	0.058	0.050	0.046
**NLCT**	0.0	0.041	0.039	0.035	0.062	0.049	0.058
	0.3	0.042	0.047	0.051	0.061	0.052	0.043
	0.6	0.044	0.062	0.047	0.052	0.049	0.042
	0.9	0.050	0.060	0.049	0.044	0.053	0.051

**Figure 1:**
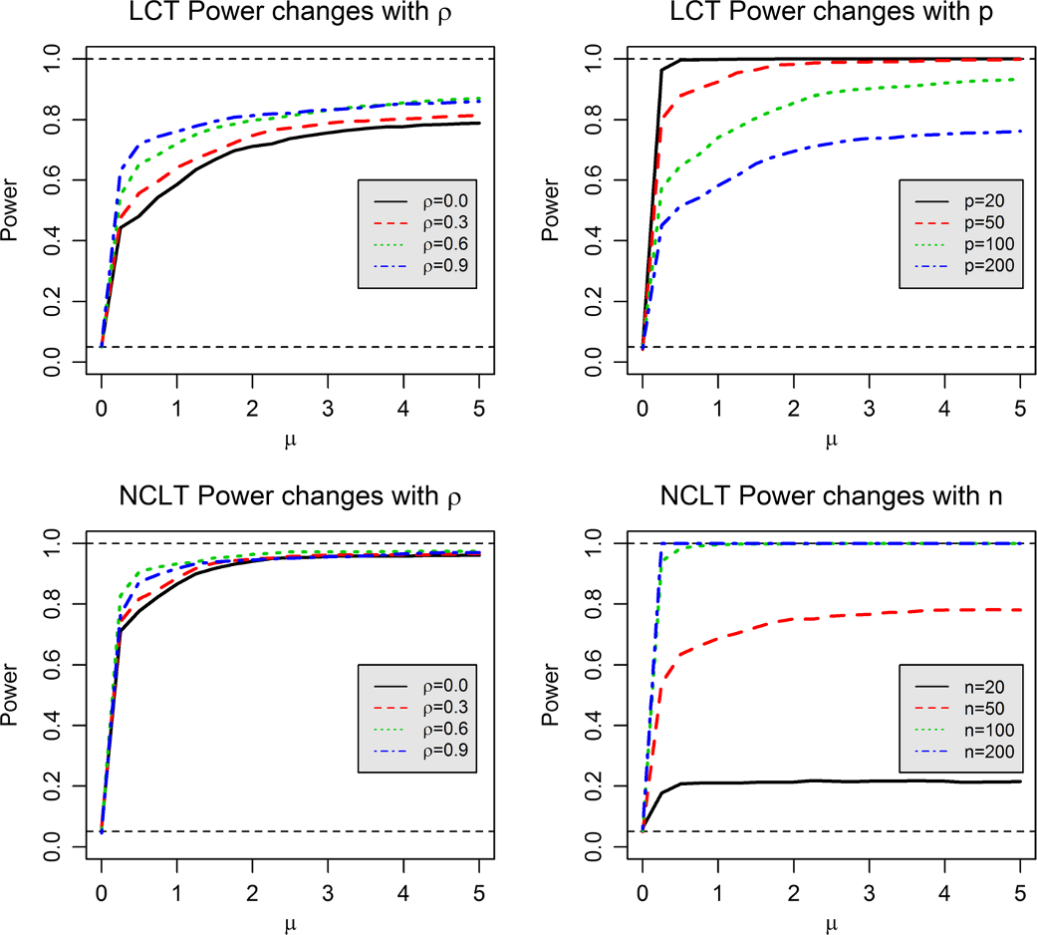
**Power changes of the two GSA methods: LCT and NLCT.**

Figure 1 illustrates the empirical power of both the LCT and NLCT methods using the nominal level of 0.05. The top left panel (*n* = 20, *p* = 20, *q* = 10 and *ρ* = 0.0/0.3/0.6/0.9) shows power change of LCT with correlation level among genes and phenotypes. At low correlation levels, LCT appears to be conservative and less powerful, which may be explained by the fact that LCT is a test based on linear combination using shrinkage approach. Intuitively, higher level of correlation between genes implies lower level of variability of the linear combination of those genes, so does the linear combination of phenotypes. Similar phenomenon can be found for NLCT method on the bottom left panel (*n* = 50, *p* = 20, *q* = 10 and *ρ* = 0.0/0.3/0.6/0.9). The top right panel (*n* = 50, p = 20/50/100/200, *q* = 10 and *ρ* = 0.6) shows power change of LCT with size of gene set. It implies that, with larger gene sets, the efficiency of LCT test drops down significantly, i.e. larger sample size is required to test larger gene sets. The bottom right panel (n = 20/50/100/200, *p* = 50, *q* = 10 and *ρ* = 0.6) shows the power change of NLCT with sample size, indicating low sample size could lead to very low power of test. Also comparing the two red lines in the right panels, we can see that NLCT is obviously less efficient than LCT when testing the linear association between genes and phenotypes.

We have two considerations for choosing *q* = 10. Firstly, the method is designed for q multiple continuous phenotypes and we wanted to show performance of LCT for a relatively large number of phenotypes, such as 10. We note that *q* = 1 reduces to our previous publication on LCT for a univariate phenotype. We reason that simulations for *q* in the range of 2 to 9 would give similar or even better performance than *q* = 10. Second, the method does not distinguish the “input” variables and “output” variables. It is in fact a correlation test, and from pure statistic point of view, there is no difference in testing results (or p-values) if one considers “X as genes and Y as phenotypes”, or if one considers “Y as genes and X as phenotypes”. Hence, our simulation scenarios with *p* = 20/50/100/200 (see upright panel of Figure 1) can also be viewed as scenarios of *q* = 20/50/100/200.

### Application

Leptin is a 16-kDa protein hormone that plays a key role in regulating energy intake and expenditure, including appetite and hunger, metabolism and behavior. It is one of the most important adipose-derived hormones [18]. Adiponectin (also refer to as GBP-28, apMI, AdipoQ and Acrp30) is a protein which in humans is encoded by *ADIPOQ* gene [19]. It is involved in regulating glucose level and fatty acid oxidation. Both leptin and adiponectin are well-known markers of human obesity [20–24]. They are hormones associated with various metabolic and inflammatory conditions. Interestingly, while leptin transcript levels are found to be over-expressed in obese subjects, adiponectin is generally under-expressed, and these may be observed not just in adipose but also in other tissues. We therefore considered using the levels of these dual markers as a multi-phenotype for application of LCT.

We applied both LCT and NLCT to analyze a real Affymetrix microarray dataset consisting of genome-wide transcriptomic measurements of prostate tumor samples from African-American prostate cancer patients [13]. The purpose of the real microarray study was to see the performance of the new approaches on testing association between gene-sets and expressions of human leptin gene (*LEP*) and adiponection gene (*ADIPOQ*). The gene expression measurements were used as surrogate phenotypes, since the blood serum measurements were not available. The publicly available data were downloaded from Gene Expression Omnibus [25] [GEO: GSE6956]. The data that we used in the present study are part of a larger microarray study of the immunobiological differences in prostate cancer tumors between African-American and European-American men. Because the *LEP* and *ADIPOQ* expression levels may be different between the two groups, we used only the data from the African-American group to examine the LCT and NLCT methods. For our analysis, we used the expression values of 13,233 genes measured in tumor samples from 33 patients. The tumor samples were resected adenocarcinomas from patients who had not received any therapy before prostatectomy and were obtained from the National Cancer Institute Cooperative Prostate Cancer Tissue Resource (CPCTR) and the Department of Pathology at the University of Maryland. According to Wallace et al. [13], the macro dissected CPCTR tumor specimens were reviewed by a CPCTR-associated pathologist who confirmed the presence of tumors in the specimens. The tissues were collected between 2002 and 2004 at four different sites. The median age of patients with prostatectomy was 61 and the median prostate-specific antigen (PSA) concentration at diagnosis was 6.1 ng/ml. Fifty-five percent of the tumors were stage pT2, and 45% were stage pT3 or more. Detailed RNA extraction, labeling and hybridization protocols were as described previously [13].

For comprehensive gene-set analysis, the C2 catalog from MsigDB [6] consisting of 1,892 gene sets were used, including metabolic and signaling pathways from major pathway databases, gene signatures from biomedical literature including 340 PubMed articles, as well as other gene sets compiled from published mammalian microarray studies. 1,846 gene sets with size range from 5 to 500 were used in our analysis. Each gene set was tested, using both LCT and NLCT approach, for its association with the *LEP* and *ADIPOQ* expression measurements.

First we run the univariate versions of LCT and NLCT for each of *LEP* and *ADIPOQ* expressions, followed by the multiple versions of LCT and NLCT for the combination of *LEP* and *ADIPOQ*, referred as a phenotype vector (*LEP, ADIPOQ*). Table 2 shows percentages of gene sets with p-values less than 0.005, 0.01, 0.05, and 0.10. We expect LCT to be more suitable than NLCT for small to moderate sample sizes. Indeed, for our application, LCT is more efficient than NLCT. For large sample size and when nonlinear relationship does exist, we expect NLCT to be more efficient than LCT. A larger percent of sets are associated with *LEP* than *ADIPOQ*. For some of the sets, the association with *LEP* is diluted by *ADIPOQ* in the multiple phenotypes analysis. However, 33 sets show a p-value smaller than 0.05 in the multiple phenotypes analysis, although their univariate analysis indicated a p-value larger than 0.05 for each of *LEP* and *ADIPOQ* phenotype (Table 3). The False Discovery Rates values based on [26] were 0.04 for *LEP*, 0.62 for *ADIPOQ*, and 0.13 for (*LEP, ADIPOQ*). The LCT and NLCT test results for all gene sets possibly associated with single phenotype *LEP/ADIPOQ* or the phenotype vector (*LEP, ADIPOQ*) were presented in the Additional file 1. Additional file 1 contains p-values and FDR-values from LCT test on gene sets for *LEP, ADIPOQ*, and *(LEP, ADIPOQ)* respectively, including all gene sets with at least one of the three p-values less than 0.05; while similar results from NLCT test were written in Additional file 2.

**Table 2:** **Percentages of gene sets with p-values less than 0.005, 0.01, 0.05 and 0.10, which from LCT/NLCT test for univariate phenotype** ***LEP*** **and** ***ADIPOQ*** **, and multiple phenotypes (** ***LEP, ADIPOQ*** **)**

Method	P-value			
	≤.005	≤.01	≤.05	≤.10
LCT for *LEP*	2.8	4.5	19.9	36.1
LCT for *ADIPOQ*	0.4	0.9	3.1	6.3
LCT for *(LEP, ADIPOQ)*	0.9	1.5	8.6	18.6
NLCT for *LEP*	0.6	1.4	7.6	16.0
NLCT for *ADIPOQ*	0.3	0.7	3.8	10.1
NLCT for *(LEP, ADIPOQ)*	0.3	0.8	5.1	11.0

**Table 3:** **Gene sets with LCT p-values for multiple phenotypes (** ***LEP*** **,** ***ADIPOQ*** **) less than 0.05, while p-values for univariate phenotype** ***LEP*** **and** ***ADIPOQ*** **are larger than 0.05**

Gene-set name	Gene-set size	*LEP* p-value	*ADIPOQ*p-value	( *ADIPOQ*, *LEP*) p-value
YEN_MYC_WT	8	0.061	0.199	0.034
GLUCONEOGENESIS	50	0.195	0.192	0.036
BYSTRYKH_HSC_BRAIN_TRANS_GLOCUS	144	0.118	0.157	0.047
PENG_LEUCINE_DN	135	0.186	0.189	0.035
AMINOSUGARS_METABOLISM	14	0.223	0.341	0.039
PENTOSE_PHOSPHATE_PATHWAY	21	0.192	0.098	0.044
ZELLER_MYC_UP	22	0.09	0.262	0.032
POMEROY_DESMOPLASIC_VS_CLASSIC_MD_DN	38	0.093	0.079	0.011
FBW7PATHWAY	8	0.088	0.314	0.048
GSK3PATHWAY	24	0.18	0.225	0.028
GOLDRATH_CELLCYCLE	31	0.21	0.105	0.048
STREPTOMYCIN_BIOSYNTHESIS	8	0.094	0.072	0.013
FRUCTOSE_AND_MANNOSE_METABOLISM	24	0.168	0.061	0.007
GLYCOLYSISPATHWAY	8	0.137	0.076	0.013
UBIQUINONE_BIOSYNTHESIS	12	0.086	0.051	0.013
HOFMANN_MANTEL_LYMPHOMA_VS_LYMPH_NODES_UP	45	0.053	0.135	0.022
HOGERKORP_CD44_DN	22	0.057	0.504	0.05
CROMER_HYPOPHARYNGEAL_MET_VS_NON_DN	72	0.11	0.178	0.05
RUTELLA_HEPATGFSNDCS_UP	144	0.058	0.136	0.045
METHOTREXATE_PROBCELL_DN	11	0.102	0.132	0.033
GENOTOXINS_4HRS_DISCR	33	0.194	0.121	0.03
HTERT_UP	57	0.083	0.089	0.036
METHOTREXATE_PROBCELL_UP	14	0.147	0.111	0.046
CAMPTOTHECIN_PROBCELL_UP	17	0.085	0.159	0.038
UV_UNIQUE_FIBRO_UP	20	0.058	0.343	0.014
CITED1_KO_HET_DN	29	0.097	0.157	0.012
HEATSHOCK_YOUNG_UP	11	0.113	0.268	0.042
HSA00051_FRUCTOSE_AND_MANNOSE_METABOLISM	35	0.148	0.075	0.044
HSA00052_GALACTOSE_METABOLISM	27	0.053	0.277	0.044
HSA00521_STREPTOMYCIN_BIOSYNTHESIS	10	0.099	0.124	0.026
HSA01030_GLYCAN_STRUCTURES_BIOSYNTHESIS_1	83	0.106	0.25	0.049
HSA04080_NEUROACTIVE_LIGAND_RECEPTOR_INTERACTION	218	0.103	0.116	0.044
HSA04120_UBIQUITIN_MEDIATED_PROTEOLYSIS	34	0.124	0.082	0.041

## Discussion

We focused here on self-contained GSA methods. We note that competitive and self-contained methods test different hypotheses, and therefore it is important for the user to make an informed choice based on the hypothesis of interest and their understanding of the limitations of the two approaches (see reviews by Nam and Kim [8] and Dinu et al. [27]). An important limitation of the self-contained approaches is that only a few genes can drive the association between the gene set and the phenotypes. In such cases, post-hoc analysis can be used to reduce the gene set to a core sub-set associated with the phenotypes. Such an analysis has been reported in simulations and in a real example for a single binary phenotype [27].

Our proposed method is useful for testing associations between sets of genes or pathways and correlated multiple continuous phenotypes. These are often measured in molecular epidemiology studies that include clinicopathological measurements of tissue features such as tumor size and staining based readouts; cellular characteristics indicated by the amount of lymphocytic infiltration in a tumor environment; and subject-specific measurements involving diagnostic or prognostic marker protein or metabolite concentrations. The LCT approach may still need to be adjusted for a mixture of continuous and categorical covariates. The *LEP* and *ADIPOQ* levels in the prostate tumor example that we have considered may also have been influenced by patient-specific covariates such as body mass index (BMI), age, and/or smoking status. We note that smoking status did not show a significant association with *LEP* expression levels (p-value = 0.36), or *ADIPOQ* expressions levels (p-value = 0.52) in our data, and BMI and age data were not available for our analysis.

The LCT approach can be used for both univariate and multivariate analyses. From the real data analysis, we can see that the univariate LCT for *LEP* is more sensitive/powerful in comparing to the multiple LCT. Generally speaking, if we knew previously that a subset of the group of phenotypes is more highly associated with the gene sets than the rest of phenotypes, then focusing on the subset of the phenotypes will gain higher power for the test, for further information is incorporated in the testing. Here, we want to point out that naively (univariately) analyzing a group of multiple correlated phenotypes will lead to problems. In the real data analysis, for controlling type I error (e.g. 0.05), it is hard to set a threshold for the two univariate tests, because of correlations between *LEP* and *ADIPOQ*. If we can assume that the two phenotypes are independent, we can set a common threshold (roughly as 0.02532057) for them. We then get 209 (11.32%) significant gene sets tested by the naïve approach, but not including 67 (3.63%) of the 159 (8.61%) significant gene sets tested by the multiple LCT. This indicates that naïve approach can identify only gene sets associated with one of the two multiple phenotypes, instead of their combination.

LCT methods rely on the linearity assumption. To check the linearity assumption, exploratory data analysis should be used prior to running a formal inference. However, a small sample size which is common in microarray studies, would limit a thorough check for nonlinearities. In the case of small sample size, we prefer using LCT instead of NLCT. The latter is suitable for relatively larger sample sizes and in the case linear assumption does not hold. Our simulation and real microarray studies indicated LCT methods perform very well for small sample sizes. The question of how small is small is debatable and depends largely on the study design. In the case of a binary/categorical phenotype, at least five samples per group are desirable. In the case of continuous phenotypes, assessing significance based on less than 10 samples is dangerous; an alternative would be to rely upon representations that are more descriptive/exploratory in nature. In terms of computation, both LCT and NLCT are highly efficient compared to other GSA methods, especially given the incorporation of the covariance matrix into the estimations.

We noticed that high correlation among genes and/or phenotypes enhances the testing power of LCT and NLCT. To understand this phenomenon, we need to distinguish correlation testing from regression modeling. In the later, we try to explain variance of the dependent variable by a group of predictors. So it is better for the predictors to be linearly independent, since high correlation among them may reduce rank(X), i.e. the real number of predictors. In the former, we are trying to find two linear combinations of genes and phenotypes respectively, with highest correlation between them. High correlation among genes may reduce p = rank(X), i.e. the real dimension of genes; and high correlation among phenotypes may reduce q = rank(Y), i.e. the real dimension of phenotypes. The smaller the dimensions p and q the easier to test the correlation between genes and phenotypes.

## Conclusions

Our proposed LCT and NLCT approaches are effective in controlling type I errors and powerful in testing associations between gene-sets and multiple continuous phenotypes. They are both computationally effective. Naively (univariately) analyzing a group of multiple correlated phenotypes, i.e., ignoring correlation structure among phenotypes, could be dangerous.

## Availability and requirements

**Project name:** Linear Combination Test for Gene-Set Analysis of Multiple Continuous Phenotypes

**Project home page:**http://www.ualberta.ca/~yyasui/homepage.html

**Operating system(s):** Microsoft Windows XP

**Programming language:** R 2.10.1

## Electronic supplementary material

Additional file 1: **Results of p-values and FDR-values from LCT test.** (XLSX 39 KB)

Additional file 2: **Results of p-values and FDR-values from NLCT test.** (XLSX 25 KB)

## Abbreviations

GSA: Gene set analysis
LCT: Linear combination test
NLCT: Nonlinear combination test.
